# Correlation of Ezrin Expression Pattern and Clinical Outcomes in Ewing Sarcoma

**DOI:** 10.1155/2017/8758623

**Published:** 2017-01-26

**Authors:** Thomas Cash, Hong Yin, Courtney McCracken, Zhi Geng, Steven G. DuBois, Bahig M. Shehata, Thomas A. Olson, Howard M. Katzenstein, Cynthia Wetmore

**Affiliations:** ^1^Department of Pediatrics, Emory University, Children's Healthcare of Atlanta, Health Sciences Research Building, Brumley Bridge, 3rd Floor, W-350, 1760 Haygood Drive, Atlanta, GA 30322, USA; ^2^Department of Pathology, Emory University, Children's Healthcare of Atlanta, 1405 Clifton Road NE, Atlanta, GA 30322, USA; ^3^Department of Pediatrics, Emory University, Children's Healthcare of Atlanta, Health Sciences Research Building, Brumley Bridge, 4th Floor, W-440-B, 1760 Haygood Drive, Atlanta, GA 30322, USA; ^4^Dana-Farber/Boston Children's Cancer and Blood Disorders Center and Harvard Medical School, 450 Brookline Avenue, Boston, MA 02215, USA; ^5^Department of Pediatrics, Emory University, Children's Healthcare of Atlanta, 1405 Clifton Road NE, Atlanta, GA 30322, USA; ^6^Department of Pediatrics, Vanderbilt University, Monroe Carell Jr. Children's Hospital at Vanderbilt, 2220 Pierce Avenue, 396c Preston Research Building, Nashville, TN 37232, USA; ^7^Department of Pediatrics, Emory University, Children's Healthcare of Atlanta, Health Sciences Research Building, Brumley Bridge, 4th Floor, W-470, 1760 Haygood Drive, Atlanta, GA 30322, USA

## Abstract

*Background*. Ezrin is a membrane-cytoskeleton linker protein that has been associated with metastasis and poor outcomes in osteosarcoma and high-grade soft tissue sarcomas. The prognostic value of ezrin expression in Ewing sarcoma is unknown.* Methods*. The relationship between ezrin expression and outcome was analyzed in a cohort of 53 newly diagnosed Ewing sarcoma patients treated between 2000 and 2011. The intensity and proportion of cells with ezrin immunoreactivity were assessed in diagnostic tumor tissue using a semiquantitative scoring system to yield intensity and positivity scores for each tumor.* Results*. Ezrin expression was detected in 72% (38/53) of tumor samples. The proportion of patients with metastatic disease was equal in the positive and negative ezrin expression groups. There was no significant difference in the 5-year event-free survival (EFS) between patients with positive versus negative ezrin expression. Patients whose tumor sample showed high ezrin intensity had significantly better 5-year EFS when compared to patients with low/no ezrin intensity (78% versus 55%; *P* = 0.03).* Conclusions*. Ezrin expression can be detected in the majority of Ewing sarcoma tumor samples. Intense ezrin expression may be correlated with a favorable outcome; however further investigation with a larger cohort is needed to validate this finding.

## 1. Introduction

Ezrin is a membrane-cytoskeleton linker protein that has pleiotropic effects on the functioning of the normal cell including directing cell polarity, motility, adhesion, invasion, and intracellular organization [1–5]. Additionally, ezrin facilitates signal transduction through adhesion molecules and a variety of growth factor receptors [3, 6]. Ezrin has been shown to play a role in tumor growth and metastasis through several mechanisms including drug efflux, prevention of apoptosis, aberrant signal transduction, and phagocytosis in certain cancers [6–12].

Increased ezrin expression has been associated with a poor prognosis in a variety of human cancers including osteosarcoma, soft tissue sarcomas (STS), breast, gastrointestinal, genitourinary, melanoma, astrocytoma, and squamous cell carcinoma of the head and neck [13–23]. An analysis performed on tumor samples from fifty patients with STS showed a significant association between positive ezrin immunoreactivity and inferior progression-free and overall survival, as well as an association with the development of distant metastasis during follow-up [24]. Similarly, in osteosarcoma, multiple studies have suggested that ezrin expression is correlated with an increased risk for recurrence and worse overall survival [6, 15, 25, 26].

Previous work in Ewing sarcoma (EWS) cell lines revealed ubiquitous, high level ezrin expression and demonstrated that the action of ezrin is dependent on the AKT/mTOR pathway [7]. MacHado et al. evaluated tumor samples from 341 patients with EWS and found that ezrin was expressed in 40.7% of the cases [27]. There have been no previous reports correlating ezrin expression with clinic characteristics and outcomes in patients with EWS. The aim of our study was to describe the patterns and frequency of ezrin expression and correlate this with clinical characteristics and outcomes in patients with Ewing sarcoma.

## 2. Materials and Methods

### 2.1. Patients

The cohort included newly diagnosed EWS patients treated at Children's Healthcare of Atlanta [(CHOA); *n* = 31] and UCSF Benioff Children's Hospital (*n* = 22) between 2000 and 2011. Any patient with diagnostic tumor tissue and relevant clinical data available were included in the study. There were no other inclusion or exclusion criteria. Formalin-fixed, paraffin-embedded surgical diagnostic biopsy samples were retrieved and used to make slides for patients treated at CHOA, while tissue for patients treated at UCSF was available in a tissue microarray. We attempted to collect information regarding* EWSR1 *translocation status in all patients. For patients with these data available, the testing had been performed using fluorescence in situ hybridization, reverse transcriptase-polymerase chain reaction, or a full karyotype analysis. All patients received alternating cycles of vincristine-doxorubicin-cyclophosphamide and ifosfamide-etoposide given on an every two- or three-week basis, in addition to local control with either surgery, radiation, or both. Institutional review board approval was obtained.

### 2.2. Immunohistochemistry

The tissue and slide preparation and immunostaining process were performed via previously described methods [28]. Tissue sections were cut at 4 *μ*m, mounted on Leica Bond Plus Slides (Cat # 00270), and air-dried at room temperature. Using the automated protocol of the Leica Bond Rx Automated Stainer (Leica Products/Equipment, Leica Microsystems, Inc., Buffalo Groove, IL), the slides were baked for 30 minutes and dewaxed with Leica Bond Dewax solution (Cat #AR9222). The antigen retrieval was Bond Epitope Retrieval 2 (Cat #AR9640), carried out in a pH 9.0 solution for 20 minutes. The anti-ezrin primary antibody dilution was 1 : 200 for 30 minutes (Cat # AB4069; Abcam Inc.). Primary antibody binding was visualized using Leica Bond Refine Detection Kit (Cat # DS9800) with a diaminobenzidine (DAB) chromogen and a hematoxylin counterstain. The negative control was prepared omitting the primary antibody. Adenocarcinoma of the colon was used as the positive control with an internal negative control (colonic mucosa). The tissue sections were independently scored in a blinded fashion by two of the study pathologists (H. Y. and B. M. S.) and were found to have at least 95% congruency.

A semiquantitative scoring system was used to quantify both ezrin positivity, that is, the percentage of cells that stained positive for ezrin, and ezrin intensity, that is, how strong the staining was in the cells. Positive expression was graded as 1+ = 1–25% of cells stained positive, 2+ = 26–50% of cells stained positive, and 3+ = 51–100% of cells stained positive. Tumors that did not express ezrin were given a positivity score of 0. Intensity of expression was graded as 1+ = weak staining, 2+ = moderate staining, and 3+ = strong staining. Tumors that did not express ezrin were given an intensity score of 0. The pattern of ezrin staining was also evaluated and was described as cytoplasmic, membranous, or cytoplasmic and membranous (diffuse).

### 2.3. Primary Predictor Variable

**Figure 1:**
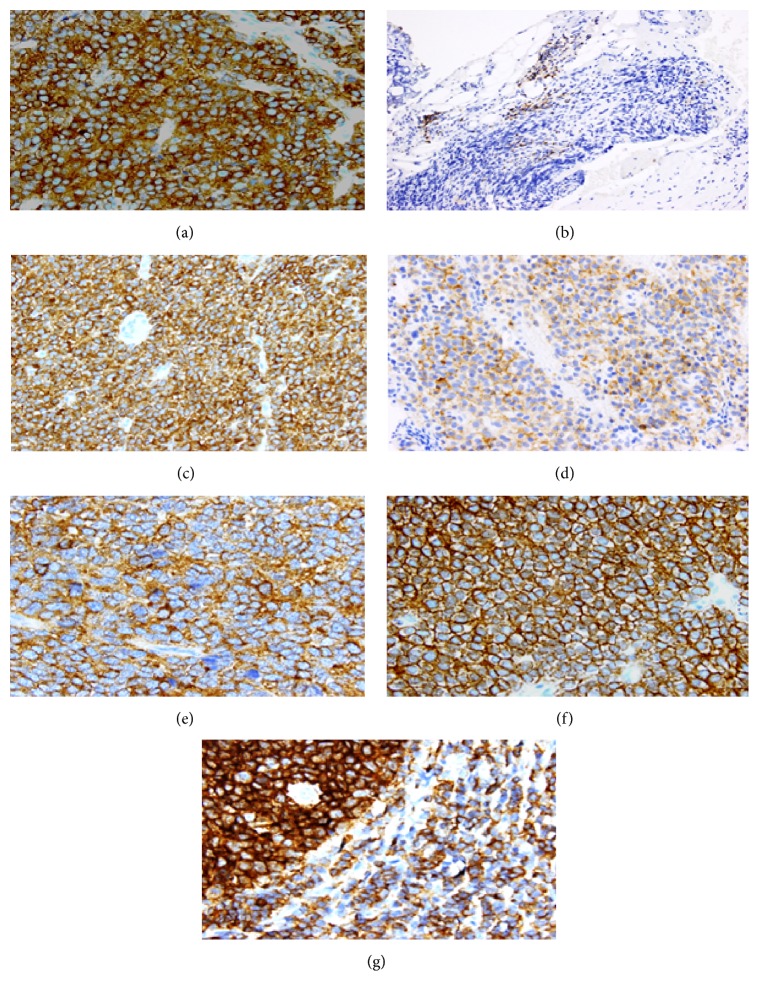
Immunohistochemistry.* Ezrin positivity*: (a) high and (b) low;* Ezrin intensity*: (c) high and (d) low;* Ezrin expression pattern*: (e) cytoplasmic, (f) membranous, and (g) diffuse.

Patients were categorized for analysis as having tumors with positive or negative ezrin expression, high (3+) versus low/no (0–2+) ezrin positivity, high (3+) versus low/no (0–2+) ezrin intensity, and cytoplasmic versus noncytoplasmic (membranous or diffuse) ezrin expression pattern (Figure 1). An ezrin composite score was created by multiplying the ezrin positivity score by the ezrin intensity score for a given patient's tumor.

### 2.4. Clinical Variables

The following clinical variables were analyzed: age; sex; race (white versus nonwhite); tumor size; primary site; and extent of disease at diagnosis (localized versus metastatic). Primary site was further categorized for analysis as either axial or nonaxial and pelvic or nonpelvic, and tumor size further categorized as ≤8 or >8 cm in maximum dimension. Tumor dimensions were obtained retrospectively from radiology reports and so were not available for all patients. Clinical outcomes of interest included death and relapse/progression.

### 2.5. Statistical Methods

Descriptive statistics were calculated for all variables of interest and included counts and percentages for categorical variables and the median and interquartile range (25th–75th) for continuous variables. Categorical variables were compared between patients with positive and negative ezrin expression, high and low/no ezrin positivity, high and low/no ezrin intensity, and cytoplasmic versus noncytoplasmic expression pattern using two-sided Fisher exact or Chi-square tests as appropriate. Continuous variables were compared between groups using the Wilcoxon rank sum test.

The primary outcome of interest was event-free survival (EFS) which was defined as the time elapsed between diagnosis and either the occurrence of an analytic event or the date of the last patient contact, whichever came first. Disease progression and death were considered analytic events. Patients who had not experienced an event as of their last contact were censored in the analysis of EFS. Overall survival (OS) was a secondary outcome and was defined as time from diagnosis to death or last follow-up for surviving patients. EFS and OS distributions were estimated by the Kaplan-Meier method. Differences in event risk between groups were evaluated using the log-rank test.

All statistical analyses were performed using SAS version 9.3 (SAS Institute, Inc., Cary, NC). Statistical significance was assessed at the 0.05 level and all *P* values are two-sided unless otherwise noted.

## 3. Results

### 3.1. Clinical Characteristics Based on Ezrin Expression

The clinical characteristics of the entire cohort are shown in Table 1. Information regarding translocation status was available from the medical records for 16/53 (30%) patients, which precluded using this variable in subset analyses. Among those whose translocation was known, 11/16 (69%) had an* EWSR1 *translocation.

Ezrin was expressed in 38/53 (72%) of the Ewing sarcoma samples in our study. The majority of these fell into the high ezrin positivity (57%) and intensity (51%) groups (Table 2). Analysis based on expression pattern showed that approximately two-thirds (68%) of the ezrin positive samples had a cytoplasmic expression pattern.

A comparison of the clinical characteristics between patients with positive versus negative ezrin expression failed to show any significant differences (data not shown). There was also no difference when the clinical characteristics were compared based on high versus low/no ezrin intensity (Table 3), high versus low/no ezrin positivity, and cytoplasmic versus noncytoplasmic expression pattern.

### 3.2. Clinical Outcomes Based on Ezrin Expression

**Figure 2:**
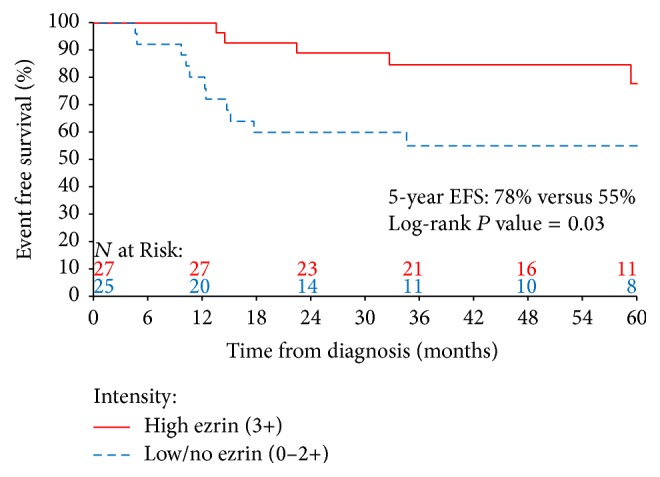
Kaplan-Meier estimates of 5-year event-free survival (EFS) for patients with tumors with high versus low/no ezrin intensity.

The 5-year EFS for each ezrin expression category (intensity, positivity, and pattern) were compared using the log-rank test. A comparison of the 5-year EFS among those with positive [65% (95% confidence interval (CI): 48%–81%)] versus negative [71% (95% CI: 46%–91%)] (*P* = 1.00), high positivity [63% (95% CI: 44%–81%)] versus low/no positivity [71% (95% CI: 50%–88%)] (*P* = 0.76), and cytoplasmic [72% (95% CI: 50%–90%)] versus noncytoplasmic [50% (95% CI: 23%–77%)] (*P* = 0.09) ezrin expression failed to show a significant difference. In contrast, the 5-year EFS for patients whose tumor showed high ezrin intensity was 78% (95% CI: 57%–93%) compared to 55% (95% CI: 35%−74%) for those with low ezrin intensity (*P* = 0.03; Figure 2). A subset analysis among patients with localized disease (*N* = 40) mirrored the results seen in the overall cohort, with patients in the high ezrin intensity group having superior 5-year EFS [86% (95% CI: 51%–96%) versus 59% (95% CI: 33%–77%); *P* = 0.02] and no significant differences among the other ezrin groups (Supplemental Table  1 available online at https://doi.org/10.1155/2017/8758623). The small number of patients with metastatic disease in our cohort limited the reliability of the survival comparisons in this group (Supplemental Table  2). We dichotomized patients at the median ezrin composite score of 6. There was no significant difference in the 5-year EFS for patients with a median ezrin composite score > 6 compared to those with a median ezrin composite score ≤ 6 (*P* = 0.14).

## 4. Discussion

Our study provides new information on the expression of ezrin in EWS and reports a novel correlation between the intensity of ezrin expression with clinical outcome. Our data show that ezrin is expressed in the majority of EWS tumor samples. We did not find any difference in the clinical characteristics between patients with an overall presence or absence of ezrin expression. There was also no difference in clinical characteristics when patients were categorized based on ezrin positivity, intensity, and expression pattern. We showed that patients whose tumors have high ezrin intensity have a superior 5-year EFS compared to patients with low or no ezrin intensity. Given the published association between ezrin expression and inferior outcomes in other sarcomas, our finding was unanticipated. We did not find a significant difference in outcomes for patients with positive versus negative ezrin expression, high versus low/no ezrin positivity, or a cytoplasmic versus noncytoplasmic ezrin expression pattern.

Similar to a prior report where high level ezrin expression was detected in 80% of EWS tumor samples, ezrin was expressed in 72% of the tumors in our study [7]. In contrast, MacHado et al. found that ezrin was expressed in only 41% of EWS tumor samples [27]. Patients in the latter study were considered as negative for ezrin expression even if low levels (5–10%) of ezrin were detected, and so this might partly explain why the incidence in this study is lower than what we have reported. Additionally, it is not known whether the tumor samples in the MacHado study were from diagnosis or from patients at the time of either surgical resection or relapse. This is important to consider as it is unclear whether ezrin expression patterns may change in response to therapy or with tumor progression. Over half (68%) of the tumor cells that were positive for ezrin expression demonstrated cytoplasmic immunoreactivity. While the pattern of ezrin expression in EWS has not previously been reported, data from osteosarcoma tumor samples showed that a cytoplasmic expression pattern occurs in 49% and was correlated with a more favorable prognosis [25]. While there was a trend towards superior EFS for patients in our study with cytoplasmic expression, this did not meet statistical significance.

For patients with high-grade STS and osteosarcoma, high ezrin expression has been correlated with inferior EFS and OS and with an increased incidence of metastasis for patients with STS [15, 24–26]. The patients in our study with positive ezrin expression were not more likely to have metastasis at diagnosis. We did not find any correlation between inferior EFS or OS and the presence or absence of ezrin expression, nor with high ezrin positivity. Krishnan et al. demonstrated that the biology of ezrin and its effects on the cell in EWS are distinct from that described in other sarcomas, and so this might partly explain these differences [7]. Paradoxically, patients with high ezrin intensity had a superior 5-year EFS compared to patients with low or no ezrin intensity. The explanation for this is unclear from our data. Most prior investigations of ezrin expression in sarcoma tumor samples have not evaluated the prognostic impact of ezrin intensity making it difficult to draw direct comparisons. It is possible that subcellular localization of the intense ezrin expression is playing a role, as ezrin is thought to be inactive in the cytoplasm [29]. A subgroup analysis comparing expression patterns by ezrin intensity failed to reveal any differences between groups, although this analysis was limited by small patient numbers (data not shown). While we did not observe any significant associations between high and low ezrin intensity groups and known EWS prognostic factors (e.g., age, stage, tumor size, and primary site) it is possible that one of these factors may be confounding our findings. It is also possible that there are other biologic differences between patients with high and low ezrin intensity that might explain the superior outcome for patients with high ezrin intensity.

This study is the first report in which ezrin expression has been correlated with clinical characteristics and outcomes in patients with EWS. While the association with clinical outcome was statistically significant, the clinical utility of this observation is not clear given the observed effect size. Our analysis was limited by the small number of available diagnostic specimens. Efforts were made to try and compare* EWSR1-ETS* fusion status with ezrin expression patterns; however, these data were only available for a small number of patients which precluded this analysis.

## 5. Conclusions

Our study shows that ezrin is expressed in the majority of Ewing sarcoma tumor samples. Intense ezrin expression may be correlated with a favorable outcome; however further studies with a larger sample size are needed to confirm this finding. In our cohort of Ewing sarcoma patients, positive ezrin expression was not correlated with a worse EFS, OS, or increased incidence of metastasis at diagnosis. Future studies should attempt to obtain paired patient samples from the time of diagnosis, surgical resection, and/or relapse to investigate whether ezrin expression patterns change over time.

## Supplementary Material

The Supplementary Material includes two tables. Supplemental Table 1 shows a comparison of the 5-year event-free survival among different ezrin expression groups for patients with localized disease, while Supplemental Table 2 shows the same comparisons for patients with metastatic disease.

## Figures and Tables

**Table 1:** Clinical characteristics of 53 patients with Ewing sarcoma.

Characteristic	Patients^1^ (*N* = 53)
Median age (25th–75th), y	13.0 (7.0–15.0)
Sex	
Male	30 (57%)
Female	23 (43%)
Race	
White	40 (76%)
Non-white	13 (25%)
Primary site	
Extremity	23 (43%)
Pelvis	11 (21%)
Chest	8 (15%)
Paraspinal	2 (4%)
Other	9 (17%)
Primary site	
Axial	31 (59%)
Nonaxial	22 (42%)
Primary site	
Pelvic	11 (21%)
Nonpelvic	42 (79%)
Tumor size, cm	
≤8	10 (19%)
>8	26 (49%)
Not available	17 (32%)
Stage	
Localized	40 (76%)
Metastatic	13 (25%)
Local control	
Surgery	12 (23%)
Radiation	14 (26%)
Surgery + radiation	5 (9%)
Not available	22 (42%)

^1^Total percentages do not sum to 100% due to rounding.

**Table 2:** Ezrin expression in 53 diagnostic Ewing sarcoma tumor samples.

Characteristic	Patients^1^ (*N* = 53)
Ezrin	
Positive	38 (72%)
Negative	15 (28%)
Ezrin positivity^2^	
0	15 (28%)
1+ (1–25%)	3 (6%)
2+ (26–50%)	5 (9%)
3+ (51–100%)	30 (57%)
Ezrin intensity^3^	
0	15 (28%)
1+ (weak)	5 (9%)
2+ (moderate)	6 (11%)
3+ (strong)	27 (51%)
Ezrin expression pattern (*N* = 38)	
Cytoplasmic	26 (68%)
Membranous	6 (16%)
Diffuse (cytoplasmic + membranous)	6 (16%)
Median ezrin composite score (25th–75th)	6 (0–9)

^1^Total percentages do not sum to 100% due to rounding.

^2^The percentage of cells that stained positive for ezrin.

^3^How strong the ezrin staining was in the cells.

**Table 3:** Comparison of clinical characteristics between patients with high and low ezrin intensity.

Characteristic	High (3+)^4^ (*N* = 27)	Low (0–2+)^4^ (*N* = 26)	*P* value
Median age (25th–75th), y	12.0 (3–15)	13.5 (11–16)	0.30^1^
Sex			0.48^3^
Male	14 (52%)	16 (62%)	
Female	13 (48%)	10 (39%)	
Race			0.69^3^
White	21 (78%)	19 (73%)	
Non-white	6 (22%)	7 (27%)	
Primary site			1.00^2^
Extremity	12 (44%)	11 (42%)	
Pelvis	5 (19%)	6 (23%)	
Chest	4 (15%)	4 (15%)	
Paraspinal	1 (4%)	1 (4%)	
Other	5 (19%)	4 (15%)	
Primary site			0.91^3^
Axial	16 (59%)	15 (58%)	
Nonaxial	11 (41%)	11 (42%)	
Primary site			0.68^3^
Pelvic	5 (19%)	6 (23%)	
Nonpelvic	22 (81%)	20 (77%)	
Tumor size, cm (*N* = 36)			0.14^3^
≤8	7 (39%)	3 (17%)	
>8	11 (61%)	15 (83%)	
Stage			0.81^3^
Localized	20 (74%)	20 (77%)	
Metastatic	7 (26%)	6 (23%)	

^1^Two-sided Wilcoxon rank sum test.

^2^Two-sided Fisher Exact test.

^3^Pearson Chi-Square test.

^4^Totals percentages do not sum to 100% due to rounding.
