# Supplementary material for: Correlation of Ezrin Expression Pattern and Clinical Outcomes in Ewing Sarcoma
Source: Sarcoma. 2017 Jan 26;2017:8758623. doi: 10.1155/2017/8758623 (PMC5299201; doi:10.1155/2017/8758623)
Supplement: Supplementary file 1 — The Supplementary Material includes two tables. Supplemental Table 1 shows a comparison of the 5-year event-free survival among different ezrin expression groups for patients with localized disease, while Supplemental Table 2 shows the same comparisons for patients with metastatic disease. [file 8758623.f1.docx]

**SUPPLEMENTAL TABLE 1** Kaplan-Meier Estimates of 5-year Event-Free Survival (EFS) for Patients with **Localized** Disease (N = 40)

| **Characteristic** | **5-year EFS** | **95% CI** | **P-value^1^** |
| --- | --- | --- | --- |
| Erin intensity |  |  |  |
| High (3+) | 86% | (51% - 96%) | 0.02 |
| Low/None (0-2+) | 59% | (33% - 77%) |  |
| Erin expression |  |  |  |
| Yes | 69% | (46% - 84%) | 0.60 |
| No | 82% | (45% - 95%) |  |
| Erin positivity |  |  |  |
| High (3+) | 68% | (43% - 84%) | 0.59 |
| Low/None (0-2+) | 77% | (43% - 92%) |  |
| Erin expression pattern |  |  |  |
| Cytoplasmic | 69% | (39% - 87%) | 0.69 |
| Non-Cytoplasmic | 67% | (28% - 88%) |  |

CI=confidence interval

^1^Log-rank test

**SUPPLEMENTAL TABLE 2** Kaplan-Meier Estimates of 5-year Event-Free Survival (EFS) for Patients with **Metastatic** Disease (N = 13)

| **Characteristic** | **5-year EFS** | **95% CI** | **P-value^1^** |
| --- | --- | --- | --- |
| Erin intensity |  |  |  |
| High (3+) | 57% | (17% - 84%) | 0.30 |
| Low/None (0-2+) | 40% | (5% - 75%) |  |
| Erin expression |  |  |  |
| Yes | 56% | (20% - 80%) | 0.21 |
| No | 33% | (<1% - 77%) |  |
| Erin positivity |  |  |  |
| High (3+) | 40% | (5% - 75%) | 0.91 |
| Low/None (0-2+) | 57% | (17% - 84%) |  |
| Erin expression pattern (N = 9) |  |  |  |
| Cytoplasmic (N=6) | 83% | (27% - 97%) | 0.01 |
| Non-Cytoplasmic (N=3) | 0% | -- |  |

CI=confidence interval

^1^Log-rank test
